# Visitor Restrictions, Palliative Care, and Epistemic Agency: A Qualitative Study of Nurses’ Relational Practice During the Coronavirus Pandemic

**DOI:** 10.1177/23333936211051702

**Published:** 2021-11-03

**Authors:** Kim McMillan, David K. Wright, Christine J. McPherson, Kristina Ma, Vasiliki Bitzas

**Affiliations:** 1 School of Nursing, 6363 University of Ottawa, Ottawa, ON, Canada; 2 Clinical Administrative Coordinator, Geriatrics and Palliative Care, CIUSSS du Centre-Ouest-de-l'Île-de-Montréal, Montreal, QC, Canada

**Keywords:** hospice care, palliative care, end of life care, COVID-19, moral agency, nursing ethics, Canada

## Abstract

Efforts to curb spread of COVID-19 has led to restrictive visitor policies in healthcare, which disrupt social connection between patients and their families at end of life. We interviewed 17 Canadian nurses providing palliative care, to solicit their descriptions of, and responses to, ethical issues experienced as a result of COVID-19 related circumstances. Our analysis was inductive and scaffolded on notions of nurses’ moral agency, palliative care values, and our clinical practice in end-of-life care. Our findings reveal that while participants appreciated the need for pandemic measures, they found blanket policies separating patients and families to be antithetical to their philosophy of palliative care. In navigating this tension, nurses drew on the foundational values of their practice, engaging in ethical reasoning and action to integrate safety and humanity into their work. These findings underscore the epistemic agency of nurses and highlight the limits of a purely biomedical logic for guiding the nursing ethics of the pandemic response.

## Introduction

The COVID-19 pandemic is drastically reshaping how healthcare is delivered and received. For patients with progressive, life-limiting illnesses and their families, physical distancing measures—and the resultant social isolation they produce—are at odds with the central role of social connection in palliative care. Harrowing media coverage of families desperately trying to connect with dying loved ones in the face of visitor restrictions throughout the pandemic continue to highlight what is at stake for patients and families (Bielsk, 2021; Reddekopp, 2020).

In this article, we report findings from a first round of data collection from a Canadian study with nurses providing palliative care to patients with progressive life-limiting illness during the COVID-19 pandemic. The impetus for this study arose after hearing disturbing stories from colleagues in palliative care, combined with ethical concerns two of us experienced in our own nursing practice, about restrictive visiting policies separating patients and their families. Virtually overnight, in-patient palliative care settings that had been designed around values of comfort, dignity, and support in dying and grieving became cold and impersonal. Spaces meant to serve relational imperatives (e.g., communal places for patients, families, and caregivers to gather and support one another), became restricted areas overtaken by biosecurity imperatives (e.g., cordoned off areas, locked doors, “do not enter” and “visitors prohibited” signs). Meanwhile, our colleagues providing palliative care in the community spoke of not being able to enter the homes of patients they had followed for many months when they were imminently dying and the abrupt cessation of many supportive services for patients and family caregivers in this setting. All of this was jarring to our ethical sensibilities around the meaning and purpose of palliative care nursing, leading us to question how the needs of dying patients and their families’ was being weighed against the risk of infection and spread of COVID-19; and if so, how were nurses navigating these end of life situations. In the following sections, we discuss relational practice in context of end of life care, highlighting how it is most often upheld and how it is disrupted in the current pandemic context, before proceeding with a description of the study methods and findings.

## Background

### Relational Practice and the Circle of Care

Palliative care philosophy is grounded in an ethos of relational practice, which is a “humanely involved process of respectful, compassionate, and authentically interested inquiry into another (and one’s own) experiences” (Hartrick Doane, 2002, p. 401). This ethos requires creativity and flexibility in its enactment (Bjornsdottir, 2018). Relational practice in palliative care nursing counters the pathologization of death and the (over)medicalization of end of life care. It is fundamentally person-centered and holistic (Browne, 2001), engaging patients and family caregivers in whatever ways best support and foster a *good death*, as defined by the patient and their loved ones (blinded for peer review, 2020).

One crucial way that relational practice toward a good death is actualized in palliative care is through the circle of care, wherein the patient is metaphorically situated at the center of a holistic approach to healthcare. This approach is responsive to physical, psychosocial, and spiritual dimensions of suffering, and supports patients’ experiences of self-determination, control, and dignity (Levine, 2000). Contributors to the circle of care first and foremost include family and may include a team of healthcare professionals (e.g., nurses, physicians, counsellors, and arts-based practitioners) as well as unregulated care providers, volunteers, and members of the communities the patient and family belong to (Bruce & Boston, 2008). Realizing the goals (existential, spiritual, and psychosocial) of care at the end of life is dependent upon the participation of the patient’s full circle of care.

Meaningful involvement of the family in care is essential to the circle-of-care concept (Randall & Downie, 2006), and nurses play a vital role in enabling such participation. The relational nature of nurses’ work and proximity to patients and families provides meaningful moments for nurses to gain unique insights into patients’ and families’ hopes, fears, goals, and needs. How nurses respond to these insights are ethically significant, as they support a good death from patients’ and families’ perspectives. Given that a patient or family’s perspective of a good death is highly individualized and changeable over time (Kehl, 2006), relational engagement with the family is required to identify—and enact—care that is consistent with the values at stake for a particular patient and family, at a particular moment in time. Nurses relational engagement might involve dialoguing with patients and families about what is happening and what to expect as the patient’s condition changes, supporting family members who are family caregivers, connecting the patient and family with other members of the team (e.g., initiating a family meeting), and helping patients and families to be together in ways that are meaningful to them in the time remaining before death. To give just one example, a nurse might recognize how overwhelming, intimidating and frightening a dying person’s body, a hospital bed, and medical equipment can appear to an anxious family member—in turn, the nurse recognizes that such angst may be lessened for a loved one by physical closeness to the dying person’s body, and might offer to help them to physically crawl into their dying loved one’s bed so they can hold them in their final moments (Ma et al., 2020).

### Disruptions in Relational Practice due to COVID-19

The circle of care has been altered, even “destabilized” (Rosa et al., 2020, p. 341) in light of the unprecedented physical distancing measures imposed in response to the COVID-19 pandemic, creating a climate that contradicts important tenets of palliative care. In the early phases of the pandemic, many palliative care programs eliminated all but the most “essential” care providers from the circle of care—meaning that while patients who were dying still had access to personal support workers, nurses, and physicians, they lost access to other providers such as volunteers, creative arts therapists, and counsellors (Negrete, 2020). Even nurses were made to limit their involvement. Community care nurses, for example, experienced policy implementation that shifted their work to virtual visits (Foulkes, 2020), except when they advocated strongly that an in-person visit was necessary. Such a priori determinations around necessity, however, inevitably limit the potential for nurses to assess and respond to emerging issues that may only become apparent from within the home (Glauser, 2020). Furthermore, the value of so-called “non-essential” care providers—or even the patient’s family themselves—as important members of the circle of care is undermined when policies do not consider them essential or necessary (Bronsther, 2020).

Perhaps the most consequential disruptions to relational practice in palliative care are the restrictions on family visiting. Policies prohibiting families and patients from being together have obvious implications for the relational experience of end of life care and for the achievement of a good death. Compounding this issue is the fact that in some palliative care settings, such policies are implemented haphazardly. An early study about the impact of COVID-19 in a sample of 16 Italian hospices, for example, showed that all had visitor restrictions, but that there was no uniformity in how these restrictions were implemented (Costantini et al., 2020). The hospices varied on whether they screened visitors before entry, the number of visitors and length of visit permitted, and the degree to which restrictions were relaxed when patient death was imminent. Similar findings were found in Taiwan, reporting 76 hospice visiting policies that varied widely during the pandemic (Hsu et al., 2020). Amidst such variability, it is difficult to discern a clear and evidence-informed logic to visitor restrictions in palliative care. This difficulty is alarming, considering the devastating consequences that such limits have for patient and family experiences of wellbeing and connectedness at the end of life (Leong et al., 2004), as well as for experiences of grief before and after death (Wallace et al., 2020). COVID-19 related visitor restrictions also have implications for end of life decision making. When substitute decision makers cannot bear witness to a patient’s decline, they may be less willing to agree to the withdrawal of aggressive treatments as death approaches, which has the potential to prolong suffering and futile care (Bronshter, 2020).

The literature is clear in that such circumstances during the COVID-19 pandemic have created moral distress for nurses (Akram, 2021; Malliarou et al., 2021; Morley et al., 2020; Wiener et al., 2021). Morley and colleagues (2020) highlight that nurses’ relationships with patients and their families during COVID-19 have been drastically impacted at end of life, tasking nurses to “temper these potentially dehumanizing scenarios of people dying alone in isolation of their loved ones” (p. 37). What is yet to be known is how nurses have mitigated morally distressing experiences resulting from the pandemic.

Nurses are indeed moral agents (Carnevale, 2013; Morley, 2018; Musto et al., 2015) who navigate moral distress in conscientious ways to cope with moral and ethical dissonance in their practice (McCarthy & Gastmans, 2015). Moral distress is navigated by nurses in a plethora of ways, which include, but are not limited to Advocating for what they believe is right (Forozeiya et al., 2019), reflecting and rationalizating on the distressing event (Thorne et al., 2018), avoiding and disengaging from morally distressing events (Forozeiya et al., 2019; Jansen et al., 2020; Thorne et al., 2018), and engaging in resistant behaviors to foster ethical reconciliation (Barlem & Ramos, 2015; Bordignon et al., 2018; Peter & Liaschenko, 2013). Given the disruptions to the landscape of palliative care nursing during the COVID-19 pandemic, our team set out to hear from palliative care nurses about the ethical issues they were experiencing in the pandemic, and how they were navigating these issues and any subsequent moral distress.

In this paper, we focus on how nurses’ ethical reasoning centers visitor restriction policies as one of the most pressing ethical issues for palliative care in the COVID-19 pandemic. It is our hope that by elevating nurses’ voices and centering their experiences in relation to this issue, we will contribute to knowledge that recognizes and validates the moral distress nurses are experiencing in the current context (Gómez-Salgado et al., 2021; Jia et al., 2020; Morley et al., 2020). Further, documentation of nurses’ perspectives about the ethics of pandemic-related policies, such as policies that restrict family visiting in end of life care, can inform the ways that relational practice imperatives are accounted for and actualized in the ongoing pandemic response.

### Methods

The research question framing this study is What ethical issues do palliative care nurses experience as a result of COVID-19 related circumstances, and how do they navigate these issues? This research question was answered qualitatively following the logic of Interpretive description, a qualitative approach designed to provide practice-relevant knowledge for clinical nursing (Thorne, 2016). Using an inductive approach, the aim of interpretive descriptive is to illuminate themes and patterns from the phenomena in foci, in a manner that can inform clinical understanding (Thorne, 2016). Epistemologically, the interpretive description approach is grounded in constructivist and naturalistic orientations in which reality is “complex, contextual, constructed, and ultimately subjective” (Thorne et al., 2004, p. 3). Interpretive descriptive is amendable to various data collection and analytic processes as long as they display philosophical coherence and a clear logic congruent to the purpose of the inquiry.

To participate in this study participants were required to be a registered nurse, registered practical nurse/licensed practical nurse, or a nurse practitioner working in any Canadian healthcare setting (e.g., hospital, hospice, long-term care, community) who self-identified as providing palliative care during the COVID-19 pandemic. Recruitment material was circulated electronically via the research teams’ professional networks as well as via social media (both Facebook and Twitter were used).

The research team consists of five researchers, all experienced in qualitative research and in the clinical nursing care of dying patients and their families. One researcher identifies as male and the remaining four, female. All researchers are registered nurses. Two hold active specialty certification in hospice palliative nursing through the Canadian Nurses Association. Three work predominantly in academia. One also maintains a current clinical practice in a residential palliative care hospice. One is a senior nursing leader in palliative care, and one is pursuing doctoral studies in Nursing. Collectively, our philosophical positioning reflects constructivism, feminism and critical theory, which lent itself to a rich theoretical lens of analysis and interpretation guided by our collective conceptual understandings of relational ethics and our own experiences as nurses providing palliative care, before and during the pandemic. Our view of relational ethics sees nurses’ abilities to transform their ethical judgement into ethical action (i.e., their moral agency), as contingent on the relationships they hold within the broader structures they inhabit (Musto et al., 2015).

Interviews were conducted online, using video-conferencing (Microsoft Teams), so that the researchers and participant could see one another during the conversation. The research team valued this method of data collection, as it offered what we believe to be much richer experiences that enabled us to still read non-verbal communication and facial expression, as opposed to phone interviews, which would have been the alternative form of data collection in light of COVID-19 social distancing measures. In our experience, we had little to no technical or internet difficulties with the exception of an occasional camera freeze, which was rectified by simply turning one or all cameras off to enhance bandwidth—none of which impacted audio. We recognize that should technical difficulties or internet delays arisen, there would be risk for data loss mid-interview.

Most interviews were conducted with two of the five researchers present, which two researchers were present varied by our availabilities. We have found that this style of interviewing allows for more in-depth exploration around areas of interest emerging in conversation. While one interviewer takes the “lead” in asking questions, the other interviewer takes note of specific ideas that merit follow up and probing. The two interviewers move back and forth over the course of the interview between these two roles (lead and note-taker), always in a conversational style and reflecting an informal tone. Having two researchers present for the interview also allows for debriefing and sharing of preliminary interpretations amongst ourselves immediately following each interview. These post-interview debriefs were recorded and served as a starting point of analysis.

Interviews were analyzed directly by the reviewing of the interview recordings numerous times by all researchers, with the aim of reflecting on the data as it related to the aims of our research and the central research question. Note taking of salient points and preliminary insights evolved into inductive thematic analysis. After multiple reviews of interview recordings independently, the team of five researchers met at multiple points both during and following data collection to discuss our thoughts and impressions of the data, which are of course, shaped by the lenses we each bring to this inquiry (constructivism, feminism, and critical theory). As interviews and ongoing analysis progressed, the impact of visitor restriction policies in in-patient settings recurred as a defining element of nurses’ experiences, challenging their moral knowing and ethical commitments to upholding palliative care philosophy in their nursing work. This paper, therefore, elaborates our findings around this specific issue, as it was the most predominant ethical issue emerging from the data. Data saturation commenced at 17 participants—it was apparent early on in the data collection that regardless of setting or category of nurse (registered nurse, registered practical nurse/licensed practical nurse, nurse practitioner), all participants were deeply challenged ethically and morally by visitor restrictions and the ensuing impacts on relational practice.

Strategies for ensuring trustworthiness of data and ongoing reflexivity included having two researchers present during the interviews, by utilizing open ended questions (for example, “Please describe any changes to your care of patients at end of life that have occurred in your setting due to COVID-19; Do you have any stories to share about specific patients who have died, since the pandemic began?”). The use of clarification questions and paraphrasing during interviews helped ensure our understandings of participants’ experiences were accurate. In-depth debriefs between researchers following each interview and utilizing a team approach to our inductive analysis allowed all for a rich and diverse interpretation of the data. We have presented the findings of this analysis to numerous nursing audiences across Canada, notably through invitations to research to practice webinars hosted by nursing associations within Canada. Each time we have received feedback that our interpretations are a faithful representation of what nurses have experienced—and continue to experience—over the course of the pandemic.

Ethics approval from the University of Ottawa (H-04-20-5736) was granted for this study. Consent forms were emailed to participants days prior to interviews and were reviewed at the commencement of the virtual interview. At that time verbal consent was received. All data was kept strictly confidential via a password protected OneDrive folder, only accessible to the research team.

### Findings

This paper reports on data collected from 17 nurse participants, from three Canadian provinces (Ontario, Alberta, and British Columbia), between June and August 2020. These participants have been practicing nursing for an average of 12 years (range, 3-32); 10 hold specialty certifications in hospice palliative care nursing from the Canadian Nurses Association. All participants were female, 1 identified as a registered practical nurse, 16 as registered nurses and 1 as a nurse practitioner. Four themes pertaining to COVID-19 visitor restricts emerged from the data, these included: *Ethical impressions of visitor restriction policies: Variable and cruel, Visit restrictions and palliative care philosophy: A clash of values, Nurses’ ethical reasoning: Integrating safety and humanity* and *Advocacy and rule breaking*. These themes will now be explained in more detail with supporting data.

#### Ethical impressions of visitor restriction policies: Variable and cruel

When nurses were asked about the most salient ethical issue they faced with the pandemic, they emphasized—consistently and without prompting—visitor restriction policies.

“The change that has impacted me the most emotionally has been the visitor restrictions…I’m finding it very hard” (Registered practical nurse, works in a hospice)

“It’s so hard to accept the situation, people are locked away from their families” (Registered nurse, works in a hospice)

Nurses recounted stories of families attempting to see loved ones amidst visitor restrictions, which left a memorable impression. For example:

“A few weeks ago, there was a resident, and his wife and kids were outside in the rain watching him sleep [through the ground-level window]….we [the nurses] were devastated” (Registered practical nurse, works in a hospice)

“One patient was admitted two weeks before she passed away. She was isolated, so no family could come and see her. By the time they could see her [after a standard post-admission quarantine period had passed] she was so delirious...she’d crawl out of bed to look for them. She didn’t understand why she was alone” (Registered nurse, works in a hospital)

Another participant described a woman nearing the end of life, her husband at the bedside, while her distraught daughters tried to connect with their mother through a hospice window.

“The women wound up dying while her kids were outside…the daughters crying and wailing, calling for their mom through a window” (Registered nurse, works in a hospice)

Visitor restrictions varied amongst and within the institutions where participants worked. Some participants working in hospital and hospice described mandatory 14-day isolation periods on admission, with no visitors permitted during that time. Outside of these mandatory isolation periods, more general restrictions were apparent, such as only allowing one “essential visitor” unless death was imminent. When the number of family members exceeded the number of allowable visitors, tough decisions had to be made:

“A patient passed away, the husband and son were there. When the third son came, one had to leave so the other could come in. That would have never happened pre-COVID, we would say ‘please, come’’’ (Registered nurse, works in a hospital)

This “tag-team” approach, as one nurse referred to it, impeded loved ones’ abilities to support each other at the bedside during these difficult moments. In one instance, the children of a patient whose wife did not speak English, translated for their mother via FaceTime from their car, as she pleaded for them to be allowed to be with her at her husband’s bedside. Another participant described a father sleeping in his vehicle in sub-zero temperatures in the hospital car park because restrictions prohibited him from being with his child.

Besides issues of access, the timing and scheduling of allowable family visits also varied. Nurses described that some settings specified a specific window of time for visiting hours, with some being as short as one hour. Other settings required pre-booking of visits, in some cases with at least 24 hours of advanced notice. Perhaps not surprisingly, participants used evocative language in describing the ethical and emotional consequence created by restrictive policies. Indeed, several participants went as far as to identify some pandemic visitation policies as *cruel.*

“[These restrictions are] robbing time, and everyone deserves that time with their family” (Registered nurse, works in a hospital)

There’s so many images in my mind of separation between family members…you feel there’s so much cruelty, separating people who are dying from their mom, or their sister” (Registered nurse, works in a hospice)

Taken together, these stories and perspectives highlight contradictions and tensions arising in contemporary palliative care practice in the context of COVID-19, including around palliative care nurses’ capacity to satisfy their ethical commitment to foster a good death for patients and families in end of life care.

“it’s distressing for staff...it all feels so cold. Working in [end of life] is about being able to provide a good death, and I find with the restrictions because of the pandemic we’re not providing the good death these people could have had” (Registered nurse, works in a hospice)

#### Visit restrictions and palliative care philosophy: A clash of values

Across our interviews, participants juxtaposed this theft of time—this cruelty—with their usual palliative care practice that centered around an ethic of family togetherness. Participants recalled how, in their role, they typically facilitate round-the-clock and “open-door” visitation, all of which was impeded in the current context.

“This policy is creating something that goes so deeply against what is my understanding and feeling of palliative care” (Registered nurse, works in a hospice)

“Family has always been a palliative intervention” (Registered nurse, works in a hospital)

Participants also expressed that the “black and white” nature of visitor policies were a further threat to their palliative care values. One of these values is person-centered care; palliative care nurses take seriously their commitment to individualizing care in response to the specific nuances that are inherent in every patient and family circumstance.

“We [palliative care providers] work within various shades of grey as opposed to black and white” (Registered nurse, works in a hospice)

“it’s [visitor restrictions] been quite hard...what I love about palliative care is that it’s very individualized...we always find a way to make something work...we make things happen” (Registered nurse, works in a hospice)

In a context of visitor restrictions, however, a “one-size-fits-all” approach superseded an individualized approach to care, creating moral distress:

“There was a one size fits all rule, which we know in palliative care, there is never a one size fits all answer and that was probably the most frustrating part” (Registered nurse, works in a hospital)

Importantly, participants felt largely left out of decisions regarding pandemic policies that had implications for their practice and for patient care. When asked about the basis for the visitation policies in their organizations, participants stated that their organization relied upon the government’s COVID-19 related public health guidelines. Some hospices, however, were not included in these guidelines. As one hospice registered nurse stated, plainly and poignantly: “We have been forgotten.”

#### Nurses’ ethical reasoning: Integrating safety and humanity

Although participants recognized the importance of public health measures to prevent the transmission of COVID-19 and the seriousness of the virus, they disagreed with the extent to which visitor restrictions and patient isolation should be happening within the context of palliative care. Participants believed that exceptions, made on a case-by-case basis, were both possible and necessary.

“In palliative…one visitor is not enough…I completely understand the context of COVID, but it feels wrong that people have to be so isolated when I do feel there are certain exceptions where we could make it work safely” (Registered nurse, works at a hospice)

Nurses’ ethical reasoning was directly influenced by their proximity to patients and families in care. This proximity created a moral weight, borne by nurses at two levels. First, at having a direct view of the suffering because of these policies. And second, by being the ones who were expected to enforce them.

“We’ve had some very, very emotional goodbyes when people get dropped off at the front of the hospice [during COVID-19], saying goodbye to their loved one for the last time, I’m getting teary-eyed just to think of it...that weight that people are experiencing when they drop their family member, they’re entering into this building and they’ll never see them again, it’s very sad” (Registered nurse, works in a hospice)

“I’m the one that had to tell a young father that he could either have his wife or his mom [be the sole visitor] and not his two children...that was one of my most heartbreaking experiences...harder than telling someone their family member has died” (Registered practical nurse, works in a hospice)

This moral weight of enforcing visitor restrictions was further evident when one participant stated:

“It’s emotionally exhausting to always be the bad guy” (Registered nurse, works in a hospice)

Being the “bad guy” also created ethical dissonance for nurses. For some participants, their reflections led them to see hypocrisy in enforcing visitor restriction policies. They explained that if the roles were reversed and it was them being kept apart from their own dying loved ones, they imagined themselves “breaking down doors” to get in.

Another source of tension for nurses was the narrow timeframe imposed by visitor policies specific to the very end of life, which allowed policy exceptions to be made only in the final hours or days before death. For nurses with expert knowledge of palliative care, this timeframe was offensive to their ethical understanding that a palliative approach should not be restricted to the last moments of life. In the last hours or days before death, many patients will have already lost the opportunity to connect with their family in meaningful ways.

“Do you really want loved ones coming to visit when the person is no longer able to speak or interact? We should be having visits earlier where they’re still capable, so it’s purposeful and meaningful for the resident and the family member” (Registered nurse, works in a hospital)

“The grounds for compassionate visiting should not be within 72 hours of end of life. If you’re terminal, that’s grounds for compassionate visiting” (Registered nurse, works in a hospice)

Participants instead pointed to other frameworks, such as the palliative performance scale (PPS) (Victoria Hospice Society, 2002), which might provide more robust structure to visitation policies than arbitrary timelines around imminent death. For example, one participant suggested that a PPS of 30 (in which a patient is totally bedbound but may still be conscious) would be a more logical and ethical threshold if the goal is to ease visitor restrictions before death. Patients with PPS scores of 30 are typically still able to engage with their loved ones in meaningful ways, and family grief is worse when such engagement does not happen.

“Palliative is not 1-2 days before death…It does so much for the dying person and the family if we get them in at a PPS of 30. Those quality conversations aren’t necessarily going to be there (if we wait until imminent death) and there might be a lot of things they need to say to each other and to be able to give them that time. When we take that piece away from families, we see them suffering later on” (Registered nurse, works in community/home care)

#### Advocacy and rule-breaking

The findings above reveal that nurses experienced moral distress as a result of visitor restriction policies they believed to be unjust. Participants utilized their moral knowing (of what is good and just) and engaged in ethical reasoning when navigating visitor restrictions. Nurses were acutely aware of what was ethically at stake should families be separated from their loved ones, and this served as their guiding compass. In response, some participants resisted policies through advocacy and rule-breaking, to safeguard palliative care values that were at stake. As one participant reflected, policies separating patients and families in palliative care has prompted “levels of advocacy I don’t think I’ve seen before” (Hospital nurse).

Participants advocated for exceptions to visitor restrictions within their institutions and at various levels of government (e.g., writing letters). Some participants described having excellent relationships with clinical directors in their workplaces. Here, nurses felt comfortable to approach these clinical directors to request exceptions based on unique family circumstances, often with success. For example, one participant described how she discussed the idea of expanding visitor access with her clinical director and was ultimately successful in having her palliative care unit exempt from the institutional visitation policy. This participant described why this change was so significant to her:

“My framework for approaching my practice is the idea of a good death, and for me, that involves family members … you can’t separate the family from palliative care” (Registered nurse, works in a hospital)

Unfortunately, other participants were not as successful in moving their moral knowing and ethical reasoning into policy changes. One participant described “pleading as much as we could...for case-by-case decision making” (Hospice nurse) to no avail, while another participant was told: “This isn’t your issue, you need to get back in your lane”; this was, as she explained, like “hitting your head against a wall and getting nowhere” (Hospital nurse).

Amidst variable leadership responses to nurses’ advocacy efforts, some participants resorted to breaking visitation rules to rectify the dissonance between their moral knowing and visitation policies. This happened when rules were deemed to interfere with whatever was ethically at stake, given the particulars of the situation. For example, nurses used their judgement when deciding how strictly to enforce requirements that family members wear personal protective equipment [PPE] and stay at least 6-feet away from each other when at the bedside of a dying loved one. When patients were isolated for potential COVID-19 exposure or pending COVID-19 testing results, nurses enforced measures such as these. But in other situations, nurses would turn a “blind eye”, to help families remove the barriers that such measures created between themselves and their loved ones. For example, if a family wanted to hold a loved one’s hand without wearing a glove, the response from the nurse might take the following form.

“I understand, just make sure in the hallway to wear your mask, and make sure you are washing your hands” (Registered nurse, works in a hospital)

The difference here is the nurse’s evaluation of risk. Nurses understood that the threat was much greater should there be a specific concern for COVID-19 spread, given whatever individual circumstances were in play. They also recognized that a broad and standardized approach to visitor restrictions carries its own *risk* for producing a bad death, which they acknowledged has severe ramifications for family members and those practitioners involved in care at end of life, including guilt and various forms of grief including disenfranchised and complicated grief. Nurses thus engaged in on-the-spot ethical reasoning, informed by their knowledge of specific patients and families.

“Has the patient been [COVID-19] swabbed? Do we know the family routine? Do we know they’re self-isolating when they’re home?” (Registered nurse, works in a hospice)

Specific examples of nurses’ resistance to visitor restriction policies recurred through the plots of individual stories that they told about caring for patients and families, at a particular time and place. These stories are meaningful because they demonstrate the ethical incommensurability of broadscale family visit prohibitions with the relational particulars of end of life moments. For example, one participant told us about working in a hospice when, early in the pandemic, a lockdown went into effect, and all visitors were expected to leave the facility at once. At this moment, the nurse allowed a daughter to stay with her father, who was imminently dying.

“If management had walked through, we would have certainly had our hand slapped for it…[but] ethically it felt wrong for that particular family at that particular moment, to tell them he had to die alone” (Registered nurse, works in a hospice)

Participants’ decisions to turn a “blind eye” or not intervene also gave recognition to families’ needs to be present and to support one another in end of life situations. For example, in realizing that one family member was not leaving the premises within an expected timeframe, given that a replacement family member had arrived, a nurse reasoned that: “This family has been here all day… I’m not kicking them out” (Hospital nurse). Another participant spoke about being in the room with family shortly after a patient had died—family members started to hug one another, which was a violation of the rule that visitors stay six feet apart.

“I would never stop a family member from hugging one another in the case of a death” (Registered nurse, works in a hospice)

Beyond choosing to not intervene, other participants told stories of actively collaborating with patients and families, to circumvent visit restriction rules. In this, participants talked about opening back stairwells for families at midnight, to provide them a few hours with their loved one in the middle of the night, away from the watchful eyes of hospital administration; or helping patients who did not smoke to venture outside for a “smoke break,” where their family would just happen to be waiting to have a short visit. Nurses ethically reasoned that circumventions such as these were justifiable after multiple requests for case-by-case exceptions for these palliative care patients had been denied by institutional leadership.

“It’s not fair to treat everybody the same, it’s not reality. Reality is that there are certain family members and situations where they are essential to the wellbeing of that person...I’m willing to have more grey, more trying to work with exceptions” (Registered nurse, works in hospice)

As described earlier, nurses’ ethical judgements in the context of pandemic-related visit restrictions involve a balance of safety, humanity and upholding the tenants of palliative care in all facets of their work. When accounting for their decisions to engage in advocacy and, in some cases, rule-breaking, nurses described a teleological reasoning process that focused on the consequences of their actions; grounding humanity as a core nursing value.

“At the end of the day, my job is to care for these patients, to provide them with the best quality of life, at the end of their life, and a big piece of that is their family...humanity comes before policy” (Registered nurse, works in a hospice)

“This isn’t necessarily about COVID-19, this is about humanity” (Registered nurse, works in community homecare)

## Discussion

Our findings reveal that COVID-19 pandemic policies that restricted family visiting were at odds with the moral imperatives that guide nurses’ relational palliative care practice. The value that families bring to palliative care cannot be overlooked, even amidst a pandemic (Bembich, 2021). Families offer an important source of moral knowledge for nurses aspiring to achieve a good death for their dying patients, particularly when the dying person is unconscious, non-verbal, or otherwise unable to engage on their own behalf (Blum & Murray, 2017). By dialoguing with the family, nurses are able to engage the personhood of the patient *through* their family. Moreover, austere visitation policies reinforce a problematic narrative in contemporary healthcare that devalues family members as stakeholders unto themselves in the circle of care (Bembich, 2021; Virani et al., 2020).

To date, COVID-19 related published literature has highlighted what is at stake when visitor restrictions are enacted, which include “undermining a desire to support family centered care” (Virani et al., 2020, p. 3) in the context of visitor restrictions for hospitalized children and resulting “relational suffering” (Bembich et al., 2021, p. 940) of parents in the context of visitor restrictions in neonatal intensive care units. The findings from our study add to this growing body of literature. Yet, even within this transformed pandemic context, nurses in our study did not waver in their commitment to helping patients and their families achieve their own good deaths in end of life care. Their perspectives thus also contribute to a narrative of moral knowing and ethical commitment in palliative and end of life nursing practice. In the following sections, we reflect on the implications of our findings for theoretical and conceptual understanding of nurses as moral agents in palliative care practice.

The ability to enact palliative care in practice is challenged by historical and administrative structures that reflect a biomedical logic; this logic represents the overarching ethos of mainstream healthcare organizations, and thus influences—sometimes in insidious ways—the policies and procedures that shape nursing work in palliative care (Glasdam et al., 2019). Thus, although visitor restrictions are “new” to the policy landscape in end of life care, in play only since the start of the pandemic, they are historically and administratively mediated; as policies are developed and implemented, certain narratives are reinforced and others silenced. The biomedical narrative of infection control has overwhelmingly dominated the pandemic response and as such, eclipsed other ways of thinking about what is *good*, *right*, and *just* in the current healthcare climate. Our findings illustrate how, against this context, nurses struggled to enact palliative philosophy in practice, which fostered experiences of moral distress. At the same time, they remained committed to both the ethical possibility and imperative of fostering good deaths for their patients. Their moral distress and ensuing resistance to visitor restriction policies contribute to a *counternarrative* (Peter et al., 2013) about the nursing ethics of COVID-19, one that is more relational than biomedical, that centers humanity, and that honours an inherent right of patients and families to be together at the end of life.

By deliberately engaging their moral knowledge about what is at stake for patients and families in end of life situations, and by advancing a humanitarian narrative grounded in a relational way of knowing, our participants’ actions—their advocacy efforts and their rule-breaking—should be understood as manifestations of *epistemic agency* (Reider, 2016). Epistemic agency is required when certain narratives—in this case, palliative care narratives—are not given “full membership in the broader epistemic community” (Townley, 2006, p. 40). This was evidenced in the ways that the knowledge of palliative care providers was seemingly not engaged in COVID-19 visitor restriction policy development and implementation. The prevailing biomedical discourse in healthcare has a longstanding history of eclipsing relational, person-based approaches (Dahl et al., 2014; Fjørtoft et al., 2021) including palliative care, with COVID-19 being no exception. Our findings serve as a reminder that relational knowledge remains underprivileged at a systems level. Nurses used epistemic agency to denounce a one size fits all approach to visitor restrictions, and to advance the agenda of palliative nursing practice. Nurses were skeptical of a one size fits all approach to visitor restrictions that, although seemingly “common sense” from an infection control perspective, interfered with the moral agenda of palliative nursing practice. Such skepticism often predisposes engagement with epistemic agency (Gómez-Alonso, 2016). Witnessing the negative impacts of visitor restrictions, nurses in our study were morally motivated to act in ways that were congruent with their moral compass, as guided by the tenets of palliative care philosophy. Nurses recognized that in certain situations in the current landscape, palliative care philosophy and the biomedical model (which epistemically drove policy) could not be reconciled, and that is was ethically necessary to align care interventions with the tenants of palliative care philosophy. Palliative care philosophy supports “flexible and non-traditional methods” (Glasdam et al., 2019, p. 141) of practice, and a one size fits all approach to visitor restrictions worked against this.

The actions of advocacy and rule-breaking served as a mechanism for making sense (“sensemaking”) and restoring order in the midst of sweeping policy changes that disrupted palliative nursing practice. According to Weick and colleagues sensemaking “starts with chaos” and serves to deal with uncertainty, with efforts more explicit when current states of existence are disrupted (Weick et al., 2005). The ability to sensemake is imperative to fostering and enacting resiliency (Powell et al., 2020), a characteristic trait required of healthcare staff during public health emergencies (Wald, 2020).

Changes in nursing practice as a result of COVID-19 are but one example that highlights pre-existing philosophical-practice gaps that occupy palliative care, and the ways in which nurses actively remedy these gaps in practice. To reiterate, these philosophical-practice gap concerns are not new to the discipline of nursing. Still, the COVID-19 pandemic response has brought to light the shortcomings in relying on biomedical logic as a predominant driver of policymaking and its far-reaching implications for care provision. Some nurses in our study acted in ways that could be perceived as resistant and irresponsible; however, literature suggests that nurse resistance can actually be a reflection of morally authentic nursing practice (Barlem & Ramos, 2015; Bordignon et al., 2018; Peter & Liaschenko, 2013). These seemingly resistant acts reflect the embodiment of relational ethical practice, that in the context of palliative care working in the “grey” is often what is needed to provide a good death. This interpretation is consistent with work by Peter et al. (2004), who suggest that acts of resistance are often a reflection of nurses’ capacity to exercise power and take ethical action. The purpose of us highlighting resistance in this paper is not to focus on the “rightness” or “wrongness” of the acts themselves, but to bring voice to the moral knowledge and ethical commitments that shape nurses' minute-to-minute decision making in their work.

### Relevance to Clinical Practice

Palliative and end of life care needs to be viewed as fundamental to healthcare provision, regardless of the setting where such care is provided, even during a pandemic. One size does not fit all in any context, including this one (Canadian Nurses Association, 2020). We are of the position that Government and healthcare institutions need to provide pandemic-related guidelines that are sensitive to palliative and end of life goals of care, and that support nurses’ case-by-case decision making. It is important that palliative approaches be systematically integrated into all areas of healthcare practice (Rosa et al., 2020; Sawatzky et al., 2016). The patients and families of the nurses who participated in our study were subject to visitor restriction policies that had little to no regard for the distinct nature of palliative care delivery across settings of care. These visitation restrictions are the antithesis of palliative and end of life care philosophy.

Arbitrary expansion of visitation rules as death becomes imminent must be re-evaluated. The exact timing of death cannot be predicted, even by the most experienced and trained healthcare professionals. Associating something as crucial as visitation at end of life to predicted time of death puts unnecessary burden on health professionals during an already extremely stressful time in their practice. Furthermore, by the time death is “imminent”, patients are often expressing very low PPS scores (Victoria Hospice Society, 2002)—experiencing wide-spread disease, are bed bound, require total care, and are drowsy, sometimes comatose, and potentially experiencing confusion and/or delirium (Victoria Hospice Society, 2002). There is inherent moral risk in postponing family visitation until such a time, and strictly limiting the number of visitors allowed.

For most older adults, death is envisioned as occurring with the comforting presence of loved ones (Thompson, 2018). Physical presence of loved ones at the time of death is strongly attributed to social understandings of a good death (Granda-Cameron & Houldin, 2012). Additionally, family separation at end of life can cause lasting psychological stress (Williams et al., 2013), and increases the potential for complicated and/or disenfranchised grief (Goveas & Shear, 2020). Visitation for families should start well before imminent death, pandemic or no pandemic. It is imperative to support authentic engagement and connection with loved ones throughout the care trajectory of terminally ill people.

### Limitations

The interpretations and conclusions drawn from this study are but one among multiple perspectives and discourses. It would be erroneous to assume that participants’ narratives necessarily capture those of all nurses. Nonetheless, similar narratives pertaining to moral distress in the context of barriers to relational practice are reflected in the wider nursing ethics literature and continue to emerge from the COVID-19 nursing literature.

The authors also wish to take the opportunity to reflect on equity and inclusion. We acknowledge that although we did not collect racial or ethnic demographic data, our sample did not accurately reflect the diversity of those in the nursing profession in Canada. This has led to important discussions and reflections amongst our research team about how best to engage racialized and visible minority healthcare professionals in palliative care nursing research, as we are acutely aware not doing so further contributes to their marginalization and lack of representation in nursing scholarship.

### Final Thoughts

As we draw this paper to a close, and reflect on the first round of data collection for this ongoing research study, we are left contemplating: can infection prevention and control logic and relational ethical logic be integrated to inform decision making and policy, and if so, how can the inherent tensions (as exemplified in this study) be reconciled? Reflecting on these questions is of utmost importance and may lead us to critically reflect upon how healthcare policy is epistemically informed, within and beyond the context of COVID-19 visitor restrictions.

## References

[bibr1-23333936211051702] Akram F. (2021). Moral injury and the COVID-19 pandemic: A philosophical viewpoint. Ethics, Medicine, and Public Health, 18, 100661. 10.1016/j.jemep.2021.100661. PMC7988441 33778145

[bibr2-23333936211051702] Barlem E. L. D. Ramos F. R. S. (2015). Constructing a theoretical model of moral distress. Nursing Ethics, 22(5), 608–615. 10.1177/0969733014551595 25366998

[bibr3-23333936211051702] Bembich S. Tripani A. Mastromarino S. Di Risio G. Castelpietra E. Risso F. (2021). Parents experiencing NICU visit restrictions due to COVID‐19 pandemic. Acta Paediatrica, 110(3), 940–941. 10.1111/apa.15620 33063339 PMC7675454

[bibr4-23333936211051702] Bielsk Z. (2021). “Hospital COVID-19 visitation rules exact a heavy toll on families, ICU staff”. https://www.theglobeandmail.com/canada/article-hospital-visitation-rules-exact-a-heavy-toll-on-families-icu-staff/

[bibr5-23333936211051702] Bjornsdottir K. (2018). “I try to make a net around each patient”: Home care nursing as relational practice. Scandinavian Journal of Caring Sciences, 32(1), 177–185. 10.1111/scs.12443 28543618

[bibr6-23333936211051702] Blum A. Murray S. J. (2017). The ethics of care: Moral knowledge, communication, and the art of caregiving. Routledge.

[bibr7-23333936211051702] Bordignon S. S. Lunardi V. L. Barlem E. L. da Silveira R. S. Ramos F. R. de Lima Dalmolin G. Barlem J. G. T. (2018). Nursing students facing moral distress: Strategies of resistance. Revista Brasileira de Enfermagem, 71(4), 1663–1670. 10.1590/0034-7167-2017-0072 30088638

[bibr8-23333936211051702] Browne A. J. (2001). The influence of liberal political ideology on nursing science. Nursing Inquiry, 8, 118-129. 10.1046/j.1440-1800.2001.00095.x 11882210

[bibr9-23333936211051702] Bruce A. Boston P. (2008). The changing landscape of palliative care: emotional challenges for hospice palliative care professionals. Journal of Hospice and Palliative Nursing, 10(1), 49-55. 10.1097/01.NJH.0000306713.42916.13

[bibr10-23333936211051702] Canadian Nurses Association . (2020). CNA’s key messages on COVID-19 and palliative care. https://cna-aiic.ca/-/media/cna/COVID-19/cna-key-messages-on-palliative-care-re-covid_e.pdf?la=en&hash=7877F640898CCCD9187DAC1DB864BA5804BBBE43

[bibr11-23333936211051702] Carnevale F. A. (2013). Confronting moral distress in nursing: Recognizing nurses as moral agents. Revista Brasileira de Enfermagem, 66 Spec, 33-38. 10.1590/S0034-71672013000700004. 24092307

[bibr12-23333936211051702] Costantini M. Sleeman K. E. Peruselli C. Higginson I. J. (2020). Response and role of palliative care during the COVID-19 pandemic: A national telephone survey of hospices in Italy. Palliative Medicine, 34(7), 889–895. 10.1177/0269216320920780 32348711 PMC7218350

[bibr13-23333936211051702] Dahl B. M. Andrews T. Clancy A. (2014). Contradictory discourses of health promotion and disease prevention in the educational curriculum of Norwegian public health nursing: A critical discourse analysis. Scandinavian Journal of Public Health, 42(1), 32–37. 10.1177/1403494813502585 23982460

[bibr14-23333936211051702] Fjørtoft A. Oksholm T. Delmar C. Førland O. Alvsvåg H. (2021). Home‐care nurses’ distinctive work: A discourse analysis of what takes precedence in changing healthcare services. Nursing Inquiry, 28(1), e12375. 10.1111/nin.12375 32725871

[bibr15-23333936211051702] Forozeiya D. Vanderspank-Wright B. Bourbonnais F. F. Moreau D. Wright D. K. (2019). Coping with moral distress – the experiences of intensive care nurses: An interpretive descriptive study. Intensive & Critical Care Nursing, 53, 23–29. 10.1016/j.iccn.2019.03.002 30948283

[bibr16-23333936211051702] Foulkes M. (2020). COVID-19 and cancer care. British Journal of Nursing, 29(10), S3. 10.12968/bjon.2020.29.10.S3 32463755

[bibr17-23333936211051702] Glasdam S. Ekstrand F. Rosberg M. van der Schaaf A. (2019). A gap between the philosophy and the practice of palliative healthcare: Sociological perspectives on the practice of nurses in specialized palliative homecare. Medicine, Healthcare, and Philosophy, 23(1), 141–152. 10.1007/s11019-019-09918-2 PMC7039838 31385188

[bibr18-23333936211051702] Glauser W. (2020). Virtual care is here to stay, but major challenges remain. Canadian Medical Association Journal, 192(30), E868–E869. 10.1503/cmaj.1095884 32719027 PMC7828921

[bibr19-23333936211051702] Gómez-Alonso M. (2016). Cartesian humility and Pyrrhonian Passivity: The ethical significance of epistemic agency. Logos & Episteme, 7(4), 461-487. 10.5840/logos-episteme20167443

[bibr20-23333936211051702] Gómez-Salgado J. Domínguez-Salas S. Romero-Martín M. Romero A. Coronado-Vázquez V. Ruiz-Frutos C. (2021). Work engagement and psychological distress of health professionals during the COVID-19 pandemic. Journal of Nursing Management, 29(5), 1016-1025. 10.1111/jonm.13239 33400325

[bibr21-23333936211051702] Goveas S. Shear K. M. (2020). Grief and the COVID-19 pandemic in older adults. The American Journal of Geriatric Psychiatry, 28(10), 1119–1125. 10.1016/j.jagp.2020.06.021 32709542 PMC7320675

[bibr22-23333936211051702] Granda-Cameron C. Houldin A. (2012). Concept analysis of good death in terminally ill patients. American Journal of Hospice & Palliative Medicine, 29(8), 632–639. 10.1177/1049909111434976 22363039

[bibr23-23333936211051702] Hsu Y. Liu Y. Lin M. Lee H. Chen T. Chou L. Hwang S. (2020). Visiting policies of hospice wards during the COVID-19 pandemic: An environmental scan in Taiwan. International Journal of Environmental Research and Public Health, 17(8), 2857–2864. 10.3390/ijerph17082857 PMC7215665 32326274

[bibr24-23333936211051702] Jansen T.-L. Hem M. H. Dambolt L. J. Hanssen I. (2020). Moral distress in acute psychiatric nursing: Multifaceted dilemmas and demands. Nursing Ethics, 27(5), 1315–1326. 10.1177/0969733019877526 31631779

[bibr25-23333936211051702] Jia Y. Chen O. Xiao Z. Xiao J. Bian J. Jia H. (2020). Nurses’ ethical challenges caring for people with COVID-19: A qualitative study. Nursing Ethics, 28(1), 33–45. 10.1177/0969733020944453 32856534 PMC7653013

[bibr26-23333936211051702] Kehl K. (2006). Moving toward peace: An analysis of the concept of a good death. American Journal of Hospice & Palliative Medicine, 23(4), 277-286. 10.1177/1049909106290380 17060291

[bibr27-23333936211051702] Leong I. Y. Lee A. O. Ng T. W. Lee L. B. Koh N. Y. Yap E. (2004). The challenge of providing holistic care in a viral epidemic: Opportunities for palliative care. Palliative Medicine. 18(1),12-18. 10.1191/0269216304pm859oa 14982202

[bibr28-23333936211051702] Levine K. (2000). The circle of care: Establishing a palliative care service in a long-term care facility. American Journal of Hospice and Palliative Medicine, 17(4), 222–223. 10.1177/104990910001700402 11883795

[bibr29-23333936211051702] Ma K. Wright D. K. Vanderspank-Wright B. Peterson W. E. Carnevale F. A. (2020). Nurses' moral experiences of ethically meaningful end-of-life care: Distress, resilience, responsibility, and care. Research and Theory for Nursing Practice, 34(3), 269–285. 10.1891/RTNP-D-19-00114. 32817280

[bibr30-23333936211051702] Malliarou M. Nikolentzos A. Papadopoulos D. Bekiari T. Sarafis P. (2021). ICU nurse’s moral distress as an occupational hazard threatening professional quality of life in the time of pandemic COVID 19. Materia Socio-Medica, 33(2), 88–93. 10.5455/msm.2021.33.88-93 34483734 PMC8385730

[bibr31-23333936211051702] McCarthy J. Gastmans C. (2015). Moral distress: A review of the argument-based nursing ethics literature. Nursing Ethics, 22(1), 131–152. 10.1177/0969733014557139 25505098

[bibr32-23333936211051702] Morley G. Grady C. McCarthy J. Ulrich C. (2020). Covid‐19: Ethical Challenges for Nurses. The Hastings Center Report, 50(3), 35–39. 10.1002/hast.1110 PMC7272859 32410225

[bibr33-23333936211051702] Musto L. C. Rodney P. A. Vanderheide R. (2015). Toward interventions to address moral distress: Navigating structure and agency. Nursing Ethics, 22(1), 91–102. 10.1177/0969733014534879 24917268

[bibr34-23333936211051702] Negrete B. (2020). Meeting the challenges of the COVID-19 pandemic: Virtual developmental music therapy class for infants in the neonatal intensive care unit. Pediatric Nursing, 46(4), 198–206.

[bibr35-23333936211051702] Peter E. Liaschenko J. (2013). Moral distress reexamined: A feminist interpretation of nurses’ identities, relationships, and responsibilities. Journal of Bioethical Inquiry, 10(3), 337–345. 10.1007/s11673-013-9456-5 23754182

[bibr36-23333936211051702] Peter E. Lunardi V. L. Macfarlane A. (2004). Nursing resistance as ethical action: Literature review. Journal of advanced nursing, 46(4), 403-416. 10.1111/j.1365-2648.2004.03008.x 15117352

[bibr37-23333936211051702] Peter E. Mohammed S. Simmonds A. (2013). Narratives of aggressive care: Knowledge, time, and responsibility. Nursing Ethics, 21(4), 461–472. 10.1177/0969733013502804 24106260

[bibr38-23333936211051702] Powell M. Froggatt K. Giga S. (2020). Resilience in in-patient palliative care nursing: A qualitative systematic review. BMJ Supportive & Palliative Care, 10(1), 79–90. 10.1136/bmjspcare-2018-001693 30808628

[bibr39-23333936211051702] Randall F. Downie R. (2006). The philosophy of palliative care critique and reconstruction. Oxford University Press.

[bibr40-23333936211051702] Reddekopp L. (2020). ‘We just want to be with him’: How COVID-19 restrictions keep families from dying loved ones. https://www.cbc.ca/news/canada/toronto/COVID-19-palliative-care-1.5517888

[bibr41-23333936211051702] Reider P. (2016). Introduction. In Reider P. (Ed.), Social epistemology and epistemic agency: Decentralising epistemic agency (pp. 21-39). Rowman & Littlefield International.

[bibr42-23333936211051702] Rosa W. Meghani S. Stone P. Ferrell B. (2020). Opportunities for nursing science to advance patient care in the time of COVID‐19: A palliative care perspective. Journal of Nursing Scholarship, 52(4), 341–343. 10.1111/jnu.12570 32725813 PMC7323245

[bibr43-23333936211051702] Sawatzky R. Porterfield P. Lee J. Dixon D. Lounsbury K. Pesut B. (2016). Conceptual foundations of a palliative approach: A knowledge synthesis. BMC Palliative Care, 15(5), 1-14. 10.1186/s12904-016-0076-9 26772180 PMC4715271

[bibr44-23333936211051702] Thompson S. (2018). “Who would want to die like that?” Perspectives on dying alone in a long-term care setting. Death Studies, 43(8), 509–520. 10.1080/07481187.2018.1491484 30207512

[bibr45-23333936211051702] Thorne S. (2016). Interpretive description: Qualitative research for applied practice (2nd ed.). Routledge.

[bibr46-23333936211051702] Thorne S. Kirkham S. R. O’Flynn-Magee K. (2004). The analytic challenge in interpretive description. International Journal of Qualitative Methods, 3(1), 1–11. 10.1177/160940690400300101

[bibr47-23333936211051702] Thorne S. Konikoff L. Brown H. Albersheim S. (2018). Navigating the dangerous terrain of moral distress: understanding response patterns in the NICU. Qualitative Health Research, 28(5), 683–701. 10.1177/1049732317753585 29357751

[bibr48-23333936211051702] Townley C. (2006). Toward a revaluation of ignorance. Hypatia, 21(3), 37-55. 10.1111/j.1527-2001.2006.tb01112.x

[bibr49-23333936211051702] Victoria Hospice Society . (2002). Palliative performance scale (PPSv2), version 2, http://www.npcrc.org/files/news/palliative_performance_scale_PPSv2.pdf

[bibr50-23333936211051702] Virani A. Puls H. Mitsos R. Longstaff H. Goldman R. Lantos J. (2020). Benefits and risks of visitor restrictions for hospitalized children during the COVID pandemic. Pediatrics (Evanston), 146(2), e2020000786. 10.1542/peds.2020-000786 32430441

[bibr51-23333936211051702] Wald H. (2020). Optimizing resilience and wellbeing for healthcare professions trainees and healthcare professionals during public health crises - practical tips for an “integrative resilience” approach. Medical Teacher, 42(7), 744–755. 10.1080/0142159X.2020.1768230 32449867

[bibr52-23333936211051702] Wallace C. Wladkowski S. Gibson A. White P. (2020). Grief during the COVID-19 pandemic: Considerations for palliative care providers. Journal of Pain and Symptom Management, 60(1), e70–e76. 10.1016/j.jpainsymman.2020.04.012 PMC7153515 32298748

[bibr53-23333936211051702] Weick K. E. Sutcliffe K. M. Obstfeld D. (2005). Organizing and the process of sensemaking. Organization Science 16(4), 409–421. 10.1287/orsc.1050.0133

[bibr54-23333936211051702] Wiener L. Rosenberg A. R. Pennarola B. Fry A. Weaver M. (2021). Navigating the terrain of moral distress: Experiences of pediatric end-of-life care and bereavement during COVID-19. Palliative & Supportive Care, 19(2), 129–136. 10.1017/S1478951521000225 33648612 PMC7985909

[bibr55-23333936211051702] Williams B. Bailey F. Woodby L. Wittich A. Burgio K. (2013). “A room full of chairs around his bed”: Being present at the death of a loved one in veterans affairs medical centers. OMEGA — Journal of Death and Dying, 66(3), 231–263. 10.2190/OM.66.3.c 23617101

